# A stress-free and easy-to-use system to expose pigs to aerosols

**DOI:** 10.1016/j.jvacx.2024.100457

**Published:** 2024-02-11

**Authors:** Jörg Jores, Nicolas Ruggli, Nadia Scalisi, Jaeyoun Jang, Sergi Torres-Puig

**Affiliations:** Institute of Veterinary Bacteriology, University of Bern, Länggassstrasse 122, CH-3012 Bern, Switzerland; Multidisciplinary Center for Infectious Diseases, University of Bern, Switzerland; Institute of Virology and Immunology IVI, Sensemattstrasse 293, CH-3147 Mittelhäusern, Switzerland; Department of Infectious Diseases and Pathobiology, Vetsuisse Faculty, University of Bern, Länggassstrasse 122, CH-3012 Bern, Switzerland; Institute of Veterinary Bacteriology, University of Bern, Länggassstrasse 122, CH-3012 Bern, Switzerland; Institute of Virology and Immunology IVI, Sensemattstrasse 293, CH-3147 Mittelhäusern, Switzerland; Department of Infectious Diseases and Pathobiology, Vetsuisse Faculty, University of Bern, Länggassstrasse 122, CH-3012 Bern, Switzerland; Institute of Veterinary Bacteriology, University of Bern, Länggassstrasse 122, CH-3012 Bern, Switzerland

**Keywords:** Pig, Aerosol delivery, 3R, Refinement, Sling

A diverse range of animal species are used in biomedical research, with mice being undoubtedly the most widely used species given their high reproductivity, well-characterized inbred lines, low costs and minimal space associated with their maintenance. Large animal species such as pigs are cost-intensive; however, they offer distinct advantages over small animals. Pigs reproduce in high numbers with a relatively short gestation period compared to ruminant livestock species, making them a very attractive alternative model available for research related to respiratory infections which feature high on the agenda of human and animal health [1], [2], [3]. Experimental airborne infections of large animal species without the need for sedation or anesthesia have been reported by different means such as the MAD Nasal™ Intranasal Mucosal Atomization Device [4], a valved mask connected to an aerosol device [5] or an infection chamber [6], [7]. The latter is commercially unavailable and requires large space, which can be problematic in high containment settings. Alternative easy-to-use systems to apply aerosols in the framework of airborne challenge models as well as for the application of aerosolized vaccines [8] or drugs [9] would be desirable to biomedical research involving the pig. We developed a stress-free system consisting of a Panepinto sling (https://www.panepinto.com) to place the pigs, a PARI LC Sprint nebulizer in combination with a PARI BOY® Classic compressor (https://www.pari.com), and an anesthesia mask with diaphragm (https://www.midmark.com) connected to the outlet of the nebulizer through the nebulizer’s mouthpiece and connective tubing. It offers relaxed breathing to the pig. The valve of the mask closes on inhalation to boost aerosol delivery and opens upon exhalation to let the air exhaust and minimize air backflow towards the nebulizer potentially interfering with the aerosol build up. A similar system has been previously reported for the Göttinger minipig [5].

In a set of experiments, we tested the aerosol size generated by the PARI BOY® Classic device and if the device can aerosolize viable bacteria of the class *Mollicutes*. Aerosols generated by the PARI LC Sprint nebulizer in combination with the PARI BOY Classic compressor into a 40L chamber had a size from 0.25 to 5 µm as detected by the aerosol spectrometer GRIMM 11-D (see Supplement). This size ensures efficient delivery of the aerosols into the lower respiratory tract. The aerosol delivery system generated *Mollicutes*-containing particles at 0.5 mL per minute. Since *Mollicutes* are cell wall-deficient bacteria that are very sensitive to shearing forces, we assumed that no drastic drop in viability of the *Mollicutes* in the output titer would be a good indication of the suitability of this device to aerosolize not only *Mollicutes* but also other pathogens that are more resistant to shearing forces, such as walled Gram-positive and Gram-negative bacteria and many viruses. First, we investigated the survival rate of the *Mollicutes* species *Mesomycoplasma hyopneumoniae* resuspended in media or buffers commonly used to generate aerosols over time. Therefore, we measured the color-changing units of serial dilutions of the different mixtures in liquid medium (Fig. 1A). Cells resuspended in phosphate buffered saline (PBS) supplemented with mucin from porcine stomach to mimic respiratory secretions that act as a physical barrier against infection from pathogens had higher survival rates over time than cells resuspended in PBS only. Then, we examined the viability of mycoplasmas upon nebulization by liquid impingement combined with subsequent serial dilutions and determination of color-changing units. Mucin (0.25 %), which is part of the mucus layer lining the respiratory tract and interacts with respiratory pathogens [10], stabilized the bacteria when added to PBS buffer compared to bacteria resuspended in PBS only (Fig. 1A).

**Fig. 1:**
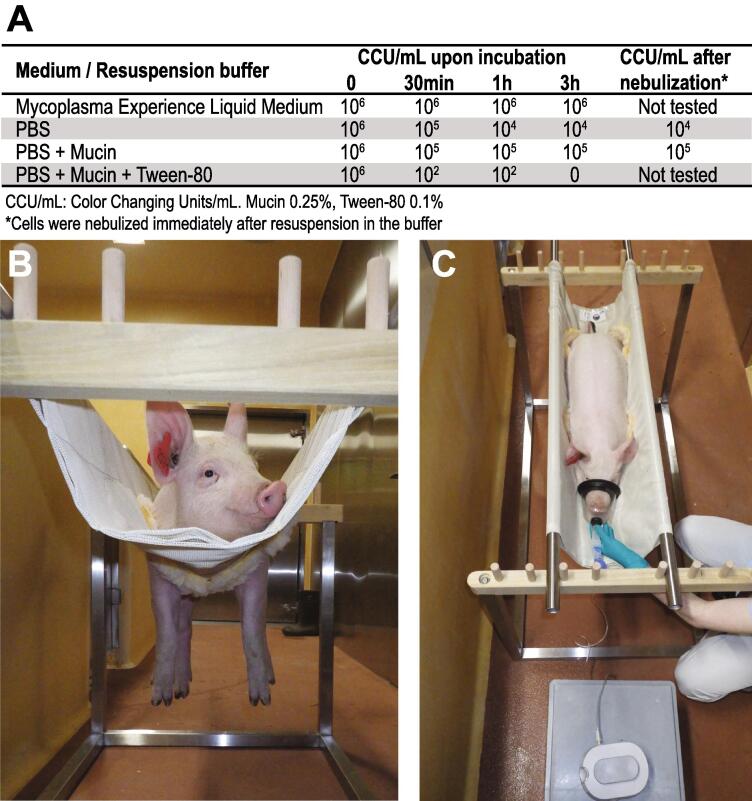
Viability of *Mesomycoplasma hyopneumoniae* in different resuspension buffers and after nebulization using the PARI LC Sprint nebulizer in combination with PARI BOY® Classic compressor (A). Pigs were conditioned to the Panepinto sling for a few minutes per day prior to aerosol exposure via a mask (B). Aerosols were channeled through a tube to the mask that was held over the snout of the animal (C).

To train the pigs to become familiar and used to the sling, we placed the pigs, 20–40 kg of body weight, on a few consecutive days into the sling for one or two minutes, which they tolerated nicely without any reactive counteraction (Fig. 1B). Finally, we placed the pigs in the sling and attached them to the mask connected to the aerosol delivery system to expose the pigs to the aerosol. Pigs tolerated the masks on their snout connected to the nebulizer and were breathing relaxed with hardly any counter movements (Fig. 1C) for up to five minutes, and longer exposure was not tested. A time of 5 min is sufficient to aerosolize 2–2.5 mL of liquid. We are convinced that such an easy-to-use model has applications for research related to infectious diseases caused by different bacteria and viruses benefiting from challenge models mirroring the natural infection. The stress-free and inexpensive system introduced here to expose pigs to aerosols fulfills the refinement of the 3R principles[11] and has a great potential to be used in the framework of other biomedical applications than only aerosol-based challenge models. Alternatively, other existing commercial nebulizers can be connected to the mask and should be compared to pick the best option for the specific needs.

This work was funded by the 10.13039/501100006454Swiss Federal Food Safety and Veterinary Office (FSVO Project No: 1.21.03) and the experiments with pigs were performed at the IVI in compliance with the animal welfare regulation of Switzerland under the cantonal license BE86/2021. Sergi Torres Puig was supported by the 10.13039/501100001711Swiss National Science Foundation (grant number 310030_201152, https://www.snf.ch). We thank Hans-Peter Lüthi, animal caretaker, for the construction of a custom device for holding and adjusting the sling.

## CRediT authorship contribution statement

**Jörg Jores:** Conceptualisation, Funding acquisition, Data curation, Writing – original draft, Writing – review and editing, Visualisation, Investigation, Validation, Formal analysis, Methodology, Supervision, Resources, Project administration. **Nicolas Ruggli:** Funding acquisition, Data curation, Writing – review and editing, Visualisation, Investigation, Validation, Formal analysis, Resources. **Nadia Scalisi:** Writing – review and editing, Investigation, Validation. **Jaeyoun Hang:** Data curation, Writing – review and editing, Visualisation, Investigation, Validation, Formal analysis, Methodology. **Sergi Torres-Puig:** Data curation, Writing – original draft, Writing – review and editing, Visualisation, Investigation, Validation, Formal analysis, Methodology.

## Declaration of competing interest

The authors declare that they have no known competing financial interests or personal relationships that could have appeared to influence the work reported in this paper.

## Data Availability

No data was used for the research described in the article.
