# Supplementary material for: A stress-free and easy-to-use system to expose pigs to aerosols
Source: Vaccine X. 2024 Feb 11;17:100457. doi: 10.1016/j.jvacx.2024.100457 (PMC10881425; doi:10.1016/j.jvacx.2024.100457)
Supplement: Supplementary data 1 [file mmc1.docx]

**Supplement**


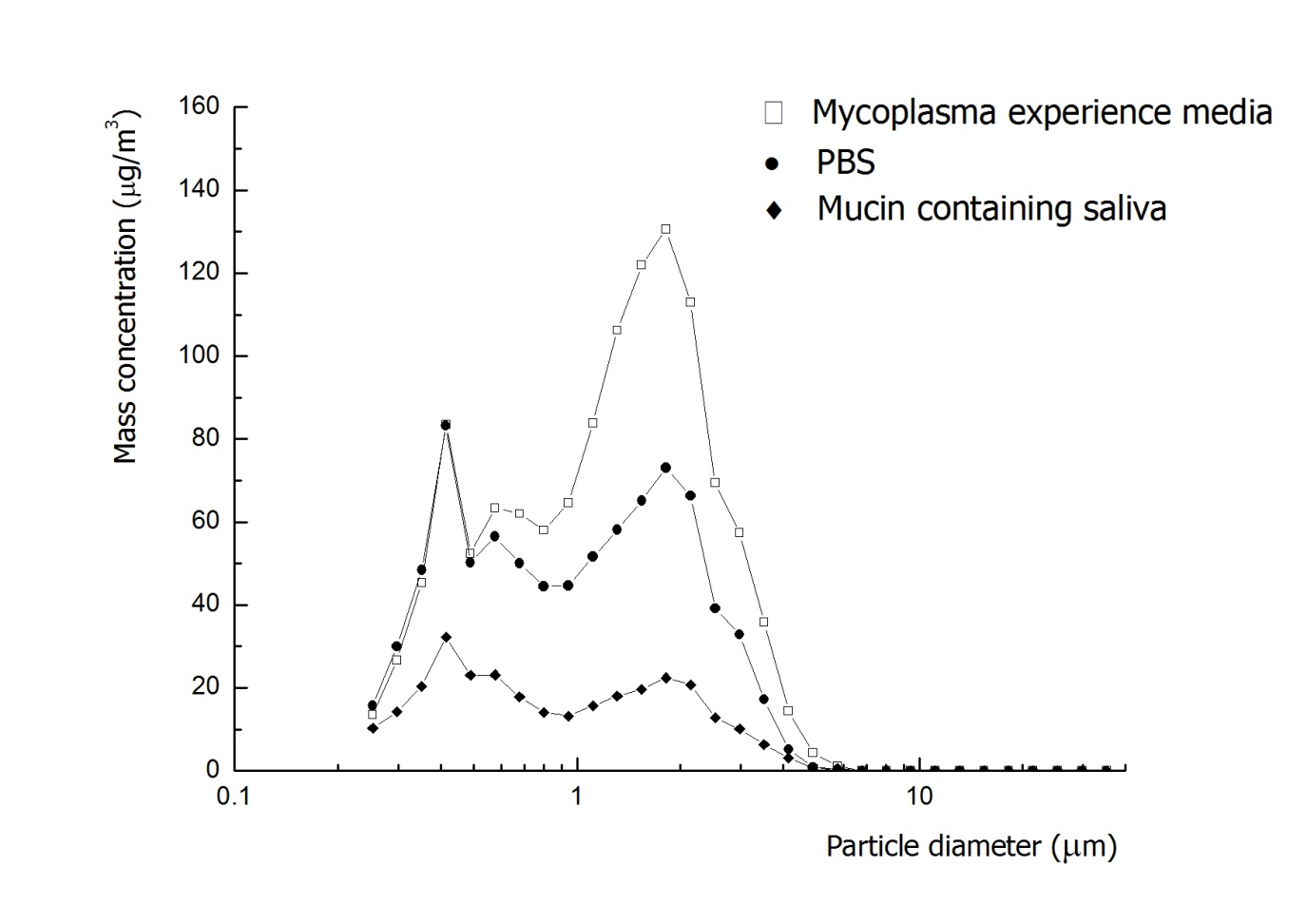


**Particle size distributions of test aerosols**

The particle size distribution and concentrations of test aerosols were studied in a 40 L chamber. The chamber was flushed with clean air using a clean air generator prior to each experiment. Mycoplasma experience liquid media, PBS, and mucin containing saliva solution were atomized into the chamber using a medical nebulizer (LC Sprint, PARI). The particle population and concentration of aerosols were monitored by aerosol spectrometer (GRIMM 11-D). The spectrometer measured particle size data over a size range of 0.25-35 µm. The aerosol sampling flow was operated in 1.2 L/min. The graph shows the particle size distribution of test aerosols at different levels of aerosol mass concentrations. The test aerosols span a size range from 0.25 to 5 µm, indicating that they are respirable and inhalable in size. We did not include *M. hyopneumoniae* in the medium for this study, as biological safety cabinet cannot accommodate the entire chamber setup. For animal experiments, it is assumed that overall particle size distribution of test aerosols containing *M. hyopneumoniae* remain unchanged, because *M. hyopneumoniae* is small in size, ranging from 0.4 to 1.2 µm.
